# A Multistage Gene Normalization System Integrating Multiple Effective Methods

**DOI:** 10.1371/journal.pone.0081956

**Published:** 2013-12-12

**Authors:** Lishuang Li, Shanshan Liu, Lihua Li, Wenting Fan, Degen Huang, Huiwei Zhou

**Affiliations:** 1 School of Computer Science and Technology, Dalian University of Technology, Dalian, Liaoning, China; 2 School of Mathmatics and Information Science and Technology, Hebei Normal University of Science and Technology, Qinhuangdao, Hebei, China; Technische Universität Dresden, Germany

## Abstract

Gene/protein recognition and normalization is an important preliminary step for many biological text mining tasks. In this paper, we present a multistage gene normalization system which consists of four major subtasks: pre-processing, dictionary matching, ambiguity resolution and filtering. For the first subtask, we apply the gene mention tagger developed in our earlier work, which achieves an F-score of 88.42% on the BioCreative II GM testing set. In the stage of dictionary matching, the exact matching and approximate matching between gene names and the EntrezGene lexicon have been combined. For the ambiguity resolution subtask, we propose a semantic similarity disambiguation method based on Munkres' Assignment Algorithm. At the last step, a filter based on Wikipedia has been built to remove the false positives. Experimental results show that the presented system can achieve an F-score of 90.1%, outperforming most of the state-of-the-art systems.

## Introduction

As a critical step of text mining in biomedical literature, gene name normalization [1] is the determination of the unique identifiers of genes and proteins mentioned in biomedical literature, so as to create the linkage between these entities and the biological databases. For example, “CARD10” may be a human gene or a mouse gene with a different gene ID in different context. “CARD10” with ID “29775” is a human gene and “CARD10” with ID “105844” belongs to a mouse gene. So they represent different types of genes and the ambiguity should be eliminated first, known as gene normalization. Once the concepts of interest are identified, more accurate information retrieval and extraction could be achieved, and more complex findings, such as relationships among entities, could be more accurately extracted. Over the past years, many solutions have been proposed for the gene name normalization task. However, despite many efforts it remains a challenging task. The main challenges for gene name normalization are as follows:

1. Gene products are often described in a phrase, rather than being referred to by a specific name.
2. Gene names are also common English words.
3. Gene and protein names often have several spelling variations or abbreviations.
4. There is the problem of inter-species gene mention ambiguity. One name or abbreviation may refer to genes in multiple species, each with its own unique ID, or even to multiple genes in the same species.

To tackle these problems, many different systems have been proposed. In general, the gene name normalization task can be decomposed into the following four components:

1. Pre-processing: detecting the named entities which denote gene names. The entities then serve as candidates with which a gene identifier may be associated. The solution for named entities detection falls roughly into two different strategies. The first strategy uses the dictionary provided by the BioCreative II organizers or an extended version of this lexicon. Hakenberg et al.'s system [2] implemented the named entity recognition process by matching the term against the synonym list. Unlike the dictionary-based approaches, the named entity taggers based on machine learning method are also employed in many systems [3]–[6]. For example, Neves et al.'s system [3] carried out a CBR-Tagger to handle the process of gene named entity recognition.
2. Dictionary matching: assigning candidate identifiers to each recognized gene mention via matching them against a lexicon. To gain more mapping pairs, many systems expanded the gene dictionaries by using additional resources or exploiting pattern-based expansion strategies. Hakenberg et al.'s system [2] expanded the provided lexical resource with additional synonyms found in EntrezGene's ‘other designations field’. And it generated lots of variants by exploiting structural, lexical, orthographical or morphological properties of gene names, such as the transformations between Latin and Arabic. Xia et al.'s system [4] collected each gene identifier's ‘description’, ‘full name from nomenclature authority’ and ‘other designations’ of EntrezGene as additional synonyms. And they made attempt to generate the token variants, such as adding lower case of synonym, replacement of punctuation with spaces. In the matching process, the exact matching and approximate matching strategies were incorporated in many systems. The core of approximate matching is to measure the similarity between the gene names in text and the terms in lexicon. Technologies for similarity computation include minimum edit distance [7], Dice coefficient [8], Jaro and JaroWinkler distance [9], etc. The Lucene package [10] is also employed as the search engine to generate the candidate list. Wermter et al.'s system [5] used the indexing and retrieval facilities of the APACHE Lucene search engine for efficient candidate retrieval. Xia et al.'s system [4] matched every gene mention occurrence with the lexical synonym through the inverted index exactly and for those unmatched gene mentions they applied a JaroWinkler similarity computation method.
3. Ambiguity resolution: determining the most appropriate identifier for the gene name when it is assigned two or more gene identifiers. The solution method falls into two main strategies. One strategy is that the disambiguation is considered as a classification task, a classifier is trained to distinguish valid gene identifiers from spurious ones. The other uses additional resources and the vector-based context models to select the most appropriate identifier among ambiguous mapping pairs. In the system of Liu et al. [6], each pair (NE, ID) was represented as a feature vector and a machine learning algorithm was used to determine the most valid pair. Hakenberg et al.'s system [11] extracted the extensive background knowledge to construct the semantic profiles for gene mention. Then the similarity metric between the context and background knowledge vector was used to distinguish valid mapping pairs from false pairs.
4. Filtering: filtering the false positives produced in the previous steps, e.g. a cell or disease name or protein family names. Many systems leaned on heuristic rules to remove potential false positives such as non-gene names, common English words or gene/protein family names. Wermter et al.'s system [5] implemented a Blacklist filter which was complied out of several public resources (such as Wikipedia, which contains several sites listing gene and protein families) to discard the unwarranted gene mentions. Hu et al.'s system [12] developed a filtering method based on the combination of the confidence scores obtained in named entity recognition, protein family names extracted from Wikipedia and the semantic similarity used in the step of disambiguation.

For BioCreative II GN task, we pay attention to two state-of-the-art systems: one is Wermter et al.'s [5], the other is Hakenberg et al.'s [11]. They both adopted the above four components and obtained good performance achieving an F-score of 86.4%, however, there is still space for further improvement.

In Wermter et al.'s system, an ML-based tagger, JNET and the dictionary-based method were combined. The tagger achieved an F-score of 80.1% performing 10-fold cross-validation on their merged corpus. Dictionary-based method could not cover all variations of gene names, so it performed ineffectively for gene names not in the training set. For the disambiguation, the context they adopted was the whole abstract (stemmed and stop words-removed), which might affect the computation of semantic similarity.

Hakenberg et al.'s system used a single finite state automaton which encoded manually regular expressions for all gene names together to recognize gene names. A gene name might not fit any regular expression in the automaton.Therefore the gene names never appearing in the training set might not be detected in the automaton. For the disambiguation, their method was based on the similarity between the gene's background knowledge (extracted from public databases) and the current abstract, and then picked the gene identifier corresponding to the highest similarity. For example, one method to calculate the similarity was based on GO terms. They searched for GO terms in the current abstract and compared them with the set of GO terms assigned to each gene candidate. The similarity was obtained from the distances of the terms in the ontology tree. In the filter, Wikipedia was not used as an external resource to filter Bio-NEs.

In this paper, we also take the four components as four subtasks and integrate them to a system for the gene name normalization task, and effective methods are proposed in each subtask. Specifically, for the gene name recognition subtask, as the fundamental step of gene normalization, we apply the gene mention tagger developed in our earlier work [13], which achieves an F-score of 88.42% on the BioCreative II GM testing set based on the two-layer stacking hybrid method. For the ambiguity resolution subtask, as the core component of gene normalization, we compute the semantic similarity based on a gene mention's context and the extended semantic information extracted from the file “gene_info” to predict the unique identifier. The gene mention's context is constructed using the entities in the abstract rather than the whole abstract. Here, Munkers' Assignment Algorithm based on JaroWinkler distance is adopted and boosts our system's performance. Furthermore, in filtering, Wikipedia, as an encyclopedia online resource, is used to remove the false positives. And combined with the approximate string matching, the filter improves F-score significantly.

The remaining part of this paper is organized as follows: the method is described in Section 2. The experiments and results are shown in Section 3. Discussion and error analysis are illustrated in Section 4 and finally, conclusions are drawn in Section 5.

## Materials and Methods

**Figure 1:**
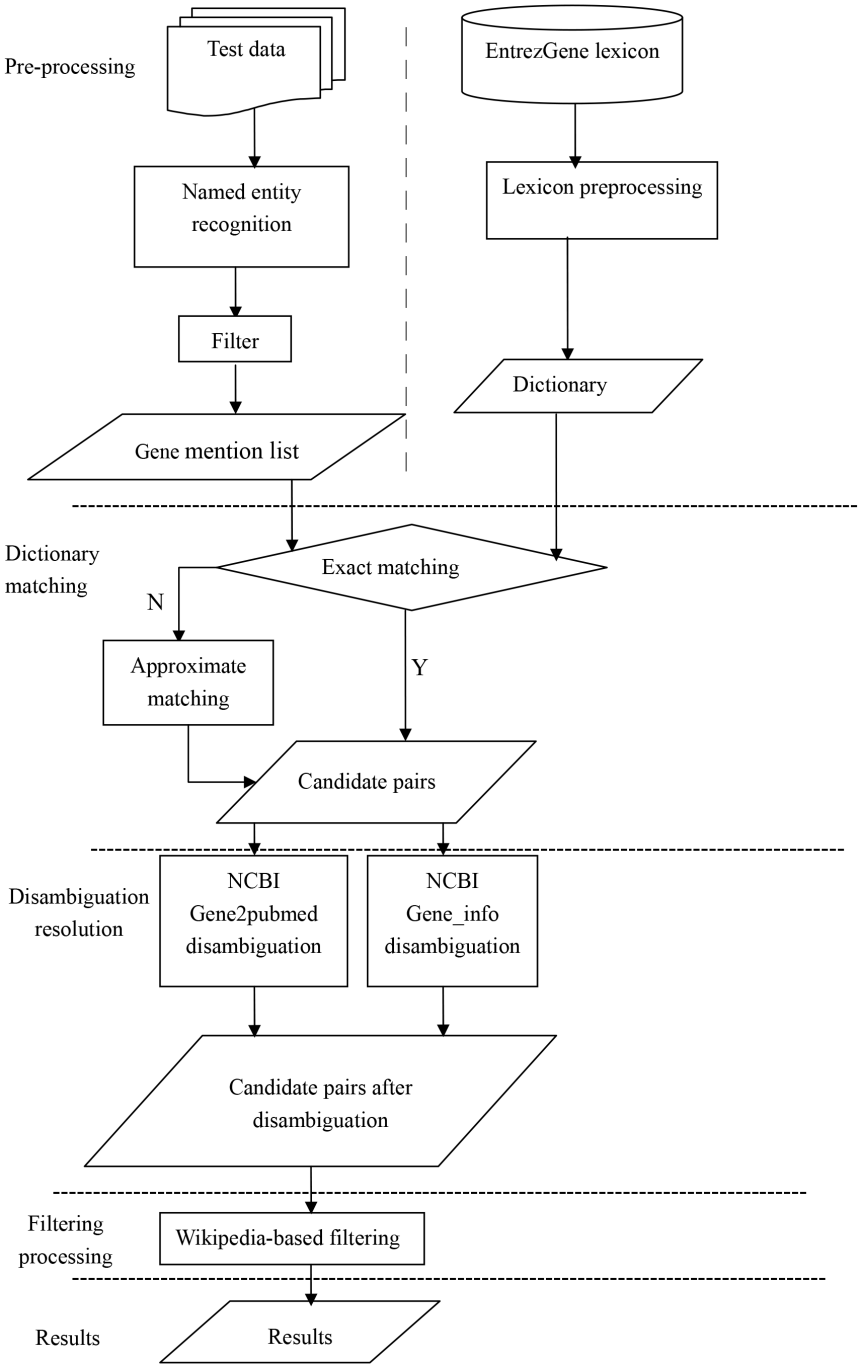
Architecture of the gene name normalization system.

The overall framework of our gene normalization system is shown Figure 1, which consists of four components. Once gene mentions in the texts have been detected, they are mapped to gene identifiers in a synonym dictionary. Subsequently, the disambiguation is implemented via a semantic similarity method, which ranks the similarity between the context and the extended semantic information of the gene identifiers. In the last step, the noisy items such as gene and protein family names are filtered based on Wikipedia.

### Pre-processing

#### Gene mention list generation

For the gene name recognition subtask, we apply a ML-tagger developed in our earlier work [13] and achieved an F-score of 88.42% on the BioCreative II GM [14] testing set. This ML-tagger was based on the two-layer stacking hybrid method which could exploit the diversity or consistency among different classifiers and the relearning process to make a final decision.

After gene name recognition, some false positives tend to be introduced. A gene family is a set of several similar genes that generally have similar biochemical functions [15]. So a gene family is not a specific gene and is not considered in the Gene Normalization task. To reduce the cost of candidate gene list generation, some false positives are removed including:

1. False positives itself

The entities ended with words like “genes”, “proteins”, “enzymes”, “receptors”, “domain”, “subunit”, “cDNA”, “mRNA”, “family”, “subfamily”, etc. will be removed. Those are gene family names rather than specific genes.

1. False positives and their contexts

The context of each false positive consists of the word before an entity or the word after an entity. For example, an initial candidate name followed by “cells” most likely refers to a cell line rather than a gene. The word “Yeast” before a candidate hints to a yeast gene instead of a human gene name. If a gene mention matches any of the above descriptions, it is removed from the list because the Gene Normalization task focuses on human genes.

#### Dictionary construction

Our dictionary is based on the original lexicon provided by BioCreative II dataset, which is composed of 32975 entries, and each entry is made up of multiple synonyms indexed by the same gene identifier. Comparing the gene mentions in abstracts with the lexicon, we find that the gene mentions have slight spelling variants and then we remove the hyphens in the lexicon. Some stop words are also removed, since they are considered to be non-informative, such as “of”, “the”, “and”.

### Dictionary matching

Here we apply a lexicon look-up method to integrate the exact string matching and approximate string matching.

#### Exact string matching

The following heuristic rules are employed to improve the coverage of the dictionary during the exact matching:

1. If a space appears in the gene mention, both the original form and the variants are considered. For example, for the term “ABC 1”, the spelling variant “ABC1”, is also put in the mapping list.
2. Roman numbers are matched with Arabic numbers. For example, for the term “ABC 1”, the spelling variant “ABC I”, also put in the mapping list.
3. The matching is case insensitive.
4. When abbreviations are introduced and a long form is followed by its abbreviation in brackets, we assign the same ID to these two kinds of candidates and vice versa.

#### Approximate string matching

**Figure 2:**
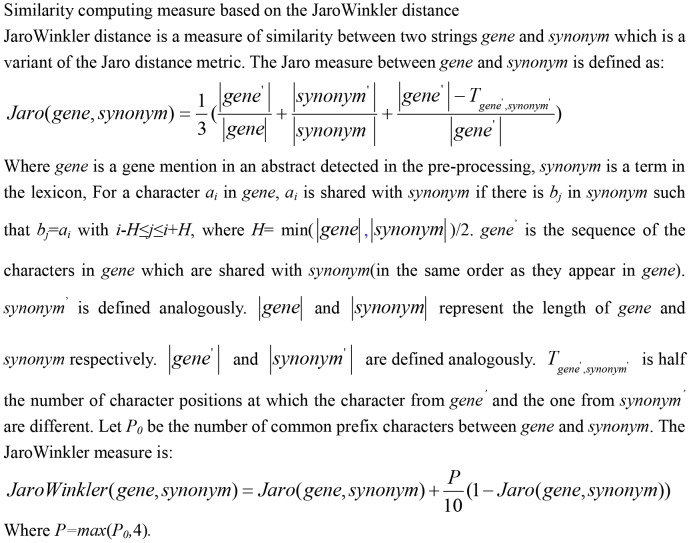
Similarity computing measure based on JaroWinkler distance.

Since the exact matching is not able to cover all of the gene mention variants, there are some genes that cannot match any entry in the dictionary. For example, the entity “serum- and glucocorticoid-induced protein kinase” is recognized in the pre-processing phase, however, it cannot get any match only by the exact matching because its form in the dictionary is “serum/glucocorticoid regulated kinase”. In order to increase the possibility of matching, the approximate string matching is combined with the exact string matching. We use an approximate matching method based on JaroWinkler distance to look up the lexicon as Figure 2.

### Ambiguation resolution

**Figure 3:**
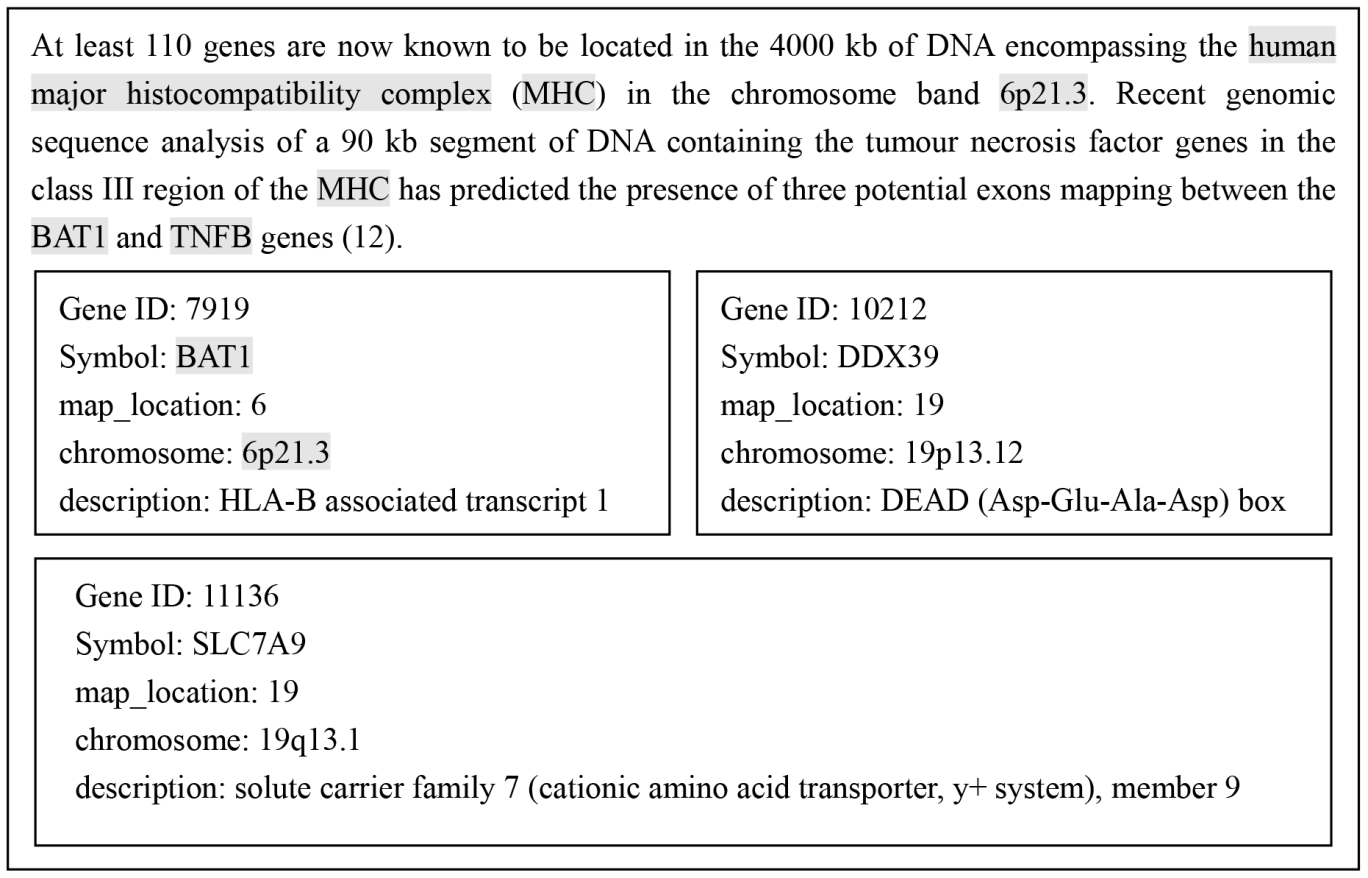
An example to determine the unique identifier based on the semantic similarity.

In the dictionary matching phase, if more than one gene identifiers can be matched by the same gene mention, these candidates need to be disambiguated. It is necessary to select the unique identifier for the gene mention that is most likely referred to in the text. In most cases, the context contains a lot of useful information to determine the unique identifier, where the context includes all Bio-NEs detected in the abstract. A representative example is given in Figure 3.

The first paragraph of the text in Figure 3 is a part from the abstract with the PubMed ID “8081366”, where the genes recognized in the preprocessing are annotated with grey shades. Take the gene “BAT1” for an example, its context consists of the genes such as “human major histocompatibility complex”, “MHC”, “6p21.3”, “BAT1” and “TNFB”. The pieces below the abstract are three identifiers of the gene “BAT1” to be disambiguated and related information extracted from the file“gene_info” provided by EntrezGene database, which we called the extended semantic information.

From the Figure 3, we can see that the gene “BAT1” is assigned three different identifiers (7919, 10212 and 11136). The correct one cannot be determined only by the gene name itself. However, in its context there is a reference to a chromosomal location “6p21.3” and Symbol “BAT1”, which points to the Gene ID “7919” out of the three identifiers most fitting the context of the gene “BAT1”, that is, the solution for the normalization of the gene name “BAT1”, as the semantic similarity between the context of the gene “BAT1” and the extended semantic information of Gene ID “7919” is the highest.

Our disambiguation method takes advantage of the extended semantic information. For the ambiguous candidate identifiers we predict the unique identifier with consideration of a gene mention's context and its identifier's extended semantic information. The gene mention's context is constructed using the entities in the abstract and the extended semantic information can be obtained by the file “gene_info”. For each gene identifier in “gene_info”, we collect the information in the four fields of ‘Symbol’, ‘chromosome’, ‘map_location’, and ‘description’. The semantic similarity between the context and the extended semantic information is calculated using JaroWinkler distance and Munkres' Assignment Algorithm [16].

JaroWinkler distance has been described in Section “Approximate string matching”, here the parameter *gene* refers to a Bio-NE in the context and the parameter *synonym* is respectively replaced with each of the four fields of “Symbol”,“chromosome”,“map_location” or “description” from the file of the extended semantic information. By calculating the JaroWinkler measure score between each gene in the context and each field in the extended semantic information, the semantic similarity between the context and the extended semantic information is obtained by summing those scores. The identifier with the highest semantic similarity is the correct identifier of the gene for disambiguation.

**Figure 4:**
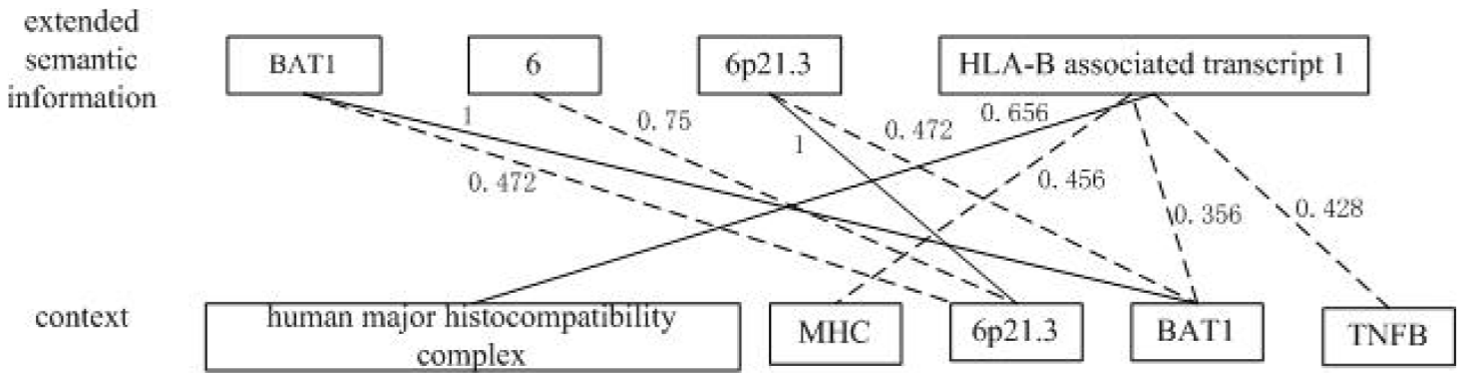
An example of the maximum matching in the bipartite graph. The maximal matching between the extended semantic information and the context is flagged with solid lines. Edges with weight 0 are bypassed and all weights are rounding in this figure.

**Figure 5:**
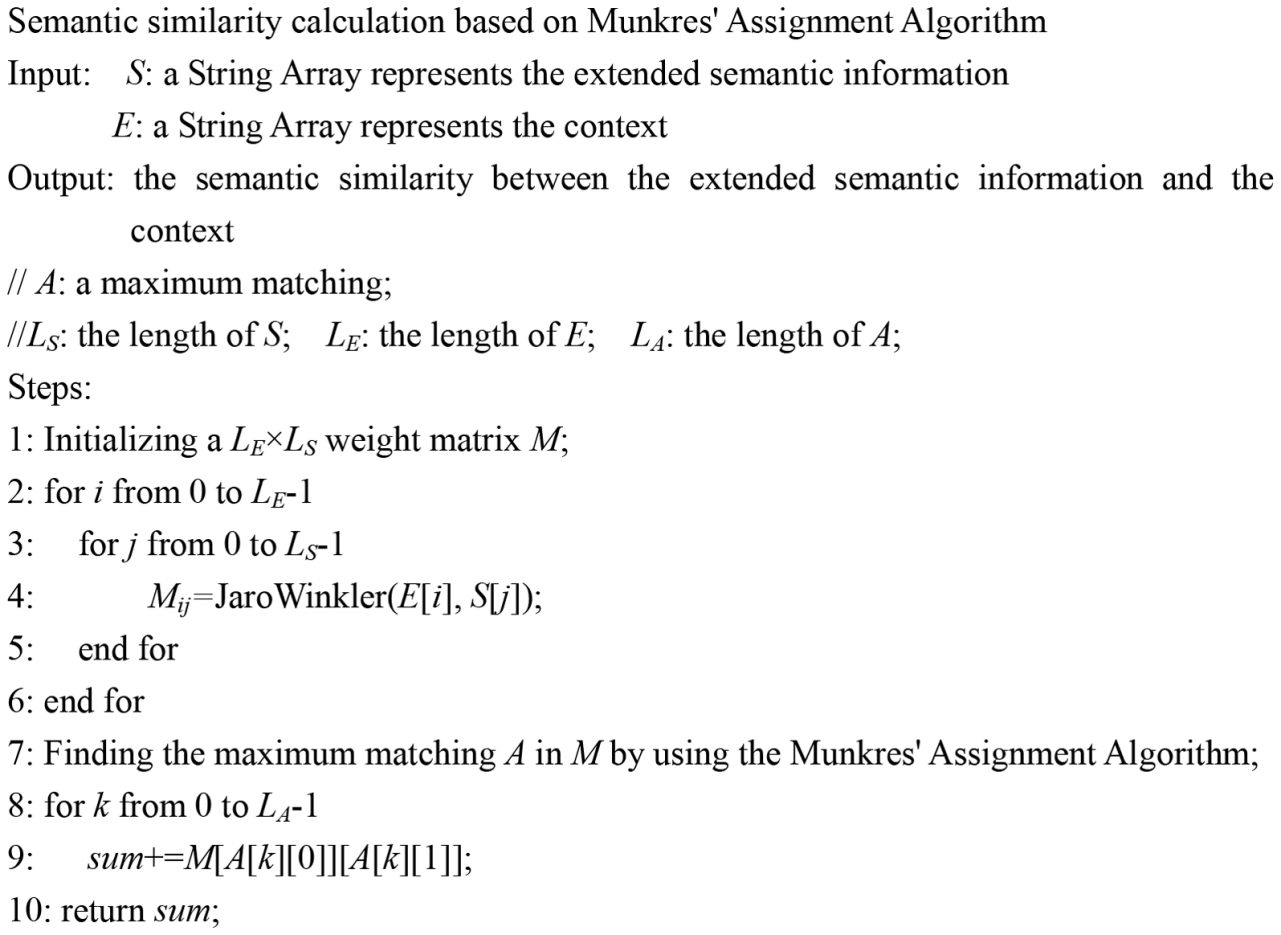
Semantic similarity calculation based on Munkres' Assignment Algorithm.

In addition, the similarity between the extended semantic information and the context depends on the matching degree of any two similar units (text fragments in the extended semantic information and in the context respectively). This process can be treated as an assignment problem in combinatorial optimization which gets the maximal matching from the global view. Munkres' Assignment Algorithm is a classical method to solve such problems. In our approach, the extended semantic information and the context are regarded as two disjoint sets *U* and *V* in a bipartite graph. All the fields in the extended semantic information and all the Bio-NEs in the context are vertices in *U* and *V* respectively. The JaroWinkler measure score is defined as the weight between a vertex in *U* and a vertex in *V*. Therefore, a weighted complete bipartite graph can be modeled. The semantic similarity can be obtained by finding the maximum matching in the bipartite graph. An example and the algorithm to calculate the semantic similarity based on Munkres' Assignment Algorithm are given in Figure 4 and Figure 5 respectively.

Additionally, the “gene2pubmed” file also provided by EntrezGene database is an annotated file which links a particular gene with PubMed ID and some gene names can be disambiguated with it.

### Filtering

Some gene family names (False Positives in our work) may be introduced in the phases of the gene name recognition and approximate string matching. Wikipedia provides a list of protein family names, we can exploit it to remove some gene family names. If a Bio-NE appears in the list, it will be removed. Different from the entities removed in Section Gene mention list generation(usually followed by the obvious words “family”, “subfamily”), the entities filtered in this section are often followed by a number, Roman numeral or Greek letter. For instance, “Zinc finger protein” is also detected as a substring in “Zinc finger protein 51”. However, the “Zinc finger protein” refers to the gene family rather than a specific gene and should be removed. In this work, a list generated from the protein family names on Wikipedia website [17] is used to filter the false positives. Every spurious gene mention in the candidate list is removed through Wikipedia filter.

## Results and Discussion

### Experimental settings

The experiments are all based on the BioCreative II dataset. The organizers provided a collection of 281 expert-annotated abstracts containing 640 gene identifiers manually mapped to human EntrezGene identifiers as training data. The test corpus consisted of 261 abstracts containing 785 identifiers manually annotated. Our system is evaluated based on the performance measures Precision and Recall supplied in the official evaluation script, and then F-score can be obtained according to Precision and Recall. The formulas are as below:
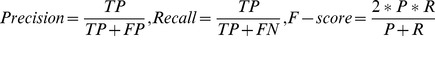
(1)where TP is true positives, FP is false positives and FN is false negatives. In this paper, the result is recognized as TP if the identifiers match the answer key, the result is recognized as FP if the identifiers do not match the answer key, and the result is recognized as FN if the gold standard identifiers do not match the answer key.

### Results

The experiments are carried out to evaluate our system's performance as follows:

1. Comparison of disambiguation methods based on different algorithms

Table 1 shows the comparison of the disambiguation methods based on different algorithms. In Table 1, both of the runs employ the filtering method based on Wikipedia. Firstly, we use the similarity scores based on JaroWinkler distance for disambiguation and achieve an F-score of 89.4%. Then Munkres' Assignment Algorithm based on JaroWinkler distance is introduced for ambiguity resolution and the F-score is 90.1%. The experiment result verifies that Munkres' Assignment Algorithm performs better. The difference between the two methods is whether there is a matching process at first. In Munkres' Assignment Algorithm, we first get the maximum matching where each edge is incident to a vertex in set *U* (a field in the extended semantic information) and a vertex in set *V* (a Bio-NE in the context), and then sum the matching edges' weights obtained from JaroWinkler distance. The sum is regarded as the final semantic similarity. While only using JaroWinkler distance for ambiguation resolution, the semantic similarity is defined as the sum of all edges' weights including those not in the matching. Therefore the semantic similarity after matching using Munkres' Assignment Algorithm is more reasonable than that directly applying JaroWinkler distance.

**Table 1:** Results based on different disambiguation algorithms.

Method	F-score	Precision	Recall	TP	FP	FN
JaroWinkler distance	89.4%	87.3%	91.5%	717	104	67
Munkres' Assignment Algorithm	90.1%	88.1%	92.1%	723	98	62

1. Comparison using the combination of different steps

Table 2 shows the results using the combination of different steps. We can see that the Recall increases from 88.4% to 92.6% while the Precision decreases by 12.7% when combining the approximate matching. Therefore the approximate matching can recall more entities for the next step (Ambiguation resolution) while some gene family names are introduced resulting in reducing the Precision. However, the gene family names can be filtered out by the Filter step. From Table 2 we can see that adding Filter the Precision (88.1%) of the combination (*Prepro.*+*Exact*+*Appro*.+*Filter*) is 12.6% higher than that of (Prepro.+Exact+Appro.), and almost the same with that (88.2%) only using the exact matching, while the Recall decreases by only 0.5%, and gets the F-Score of 90.1%. In short, combined with the approximate matching, the system can recall more True Positives and remove some False Positives by the Filter and finally obtain the best performance.

**Table 2:** Results using the combination of different steps.

Method	F-score	Precision	Recall	TP	FP	FN
Prepro.+Exact	88.3%	88.2%	88.4%	694	93	91
Prepro.+Exact+Appro.	83.2%	75.5%	92.6%	726	236	58
Prepro.+Exact+Appro.+Filter	90.1%	88.1%	92.1%	723	98	62

*Prepro.* is short for pre-processing; *Exact* stands for Exact string match; *Appro.* means Approximate string matching and *Filter* is based on Wikipedia.

1. Comparison with other systems

We also compare our results with some systems for BioCreative II GN task in Table 3. It shows that our method is comparable to the current state-of-the-art systems in this task. We get an F-score of 90.1%(Precision:88.1% Recall:92.3%) on the BioCreative II gene normalization test data. Compared with Wermter et al.'s and Hakenberg et al.'s systems, the main different aspects are the methods of the gene mention recognition and disambiguation:

**Table 3:** Comparison with other systems.

Method of authors	Precision	Recall	F-score	TP	FP	FN
Our system	88.1%	92.3%	90.1%	723	98	62
Wermter et al., 2009[5]	87.8%	85.0%	86.4%	668	76	118
Hakenberg et al., 2008[11]	90.7%	82.4%	86.4%	647	66	138
Hu et al., 2012[12]	83.5%	82.5%	83.0%	648	128	137

For the gene mention list generation, Wermter et al.'s system combined a tagger, JNET, based on CRF and a dictionary to detect gene names. The tagger achieved 80.1% F-score performing a 10-fold cross-validation on their organized training set. And the method based on dictionary was limited by the size of dictionary and could not perform well on the gene names not in the dictionary. In Hakenberg et al.'s system, regular expressions for gene names were encoded together in a single finite state automaton. A gene name might not fit any regular expression in the automaton and could not be detected. In our system, a two-layer stacking hybrid method [13] is exploited and achieved 88.42% F-score on the BioCreative II GM testing data, which outperforms most of the state-of-the-art systems.

In the phase of ambiguation resolution, the gene context is used to decide the correct identifier. In Wermter et al.'s system, they constructed the background knowledge for all the identifiers in the dictionary and Semantic Profile Index for background knowledge was created using Lucene. The semantic similarity was computed by querying the whole abstract (stemmed and stop word-removed) text in which a gene mention to be disambiguated occurred against the Semantic Profile Index. Though the abstract was stemmed, with stop words removed, it might include some noisy information resulting in inadequate semantic similarity scores. In our work, the context includes all the Bio-NEs occurring in the abstract instead of the whole abstract so that lots of noisy information can be ignored. As the performance to recognize the Bio-NEs is satisfactory, such context is helpful to the similarity measure. Besides, Munkers' Assignment Algorithm based on JaroWinkler distance contributes to our system's performance.

Furthermore, the combination of the approximate string matching and the filter based on Wikipedia improves F-score significantly.

### Discussion

From the above comparison of the experiment results, we discuss our system's effectiveness as follows:

1. Effectiveness of the Bio-NEs recognition

We incorporate our powerful NER system [8] into the gene normalization system. The tagger is based on the two-layer stacking method. Six different classifiers are respectively trained at layer-0. Then at layer-1, the results of single classifiers are regarded as feature vectors and the stacking result can be obtained from the CRF. The tagger achieves 88.42% F-score on the BioCreative II GM testing data. The two-layer stacking method performs better as: (I)It can exploit the diversity or consistency among different classifiers to make a final decision on the basis of single models. (II) It has the capability of relearning from the original learning at layer-0. Only the better the performance of recognition is, can the gene normalization performs better.

1. Effectiveness of the ambiguation resolution method

We employ the useful context for disambiguation. The context in our system ignores lots of noisy information and is helpful to the similarity measure on account of the satisfactory Bio-NEs recognition performance. Besides, Munkers' Assignment Algorithm improves the measure of semantic similarity and boosts our system's performance.

1. Effectiveness of the combination of the approximate string matching and the Filter based on Wikipedia

It can be seen from the experiment results (*Prepro.*+*Exact*+*Appro.*) in Table?>;2 that more gene names can get candidate identifiers by the approximate string matching, and therefore the recall increases significantly. Though this step may introduce some wrong pairs (NE, identifier) resulting in the precision's decrease, after the filter based on Wikipedia, the system removes some False Positives and finally obtain better performance (the experiment result (*Prepro.*+*Exact*+*Appro.*+*Filter*)). The approximate string match improves the recall and the Filter based in Wikipedia contributes to the precision respectively. Finally, their combination enhances the system's performance.

### Error analysis

**Figure 6:**
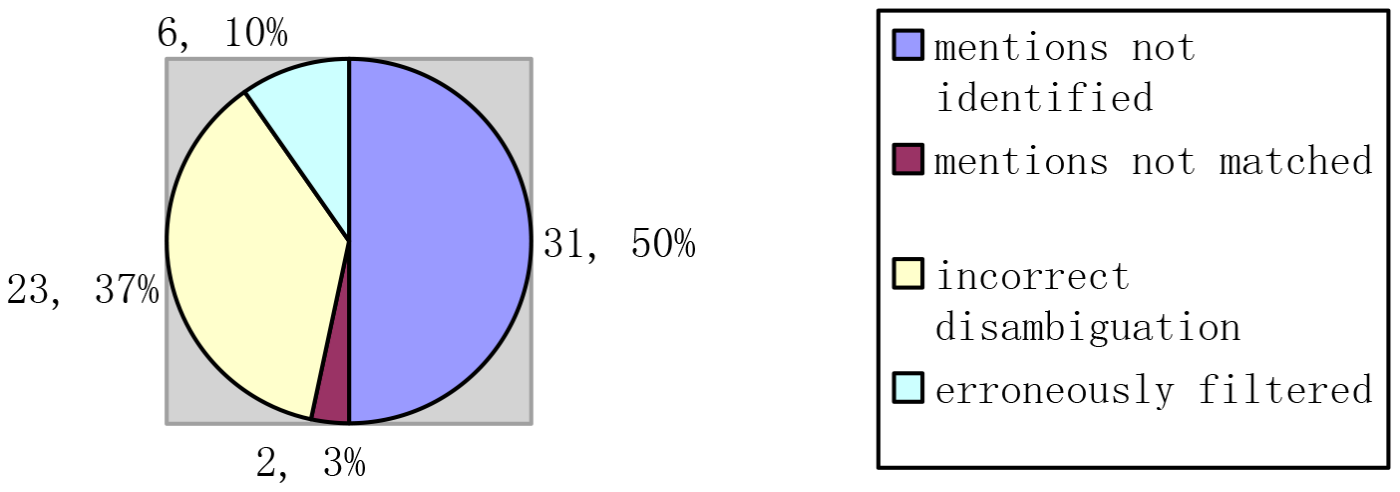
Causes for FN errors.

**Figure 7:**
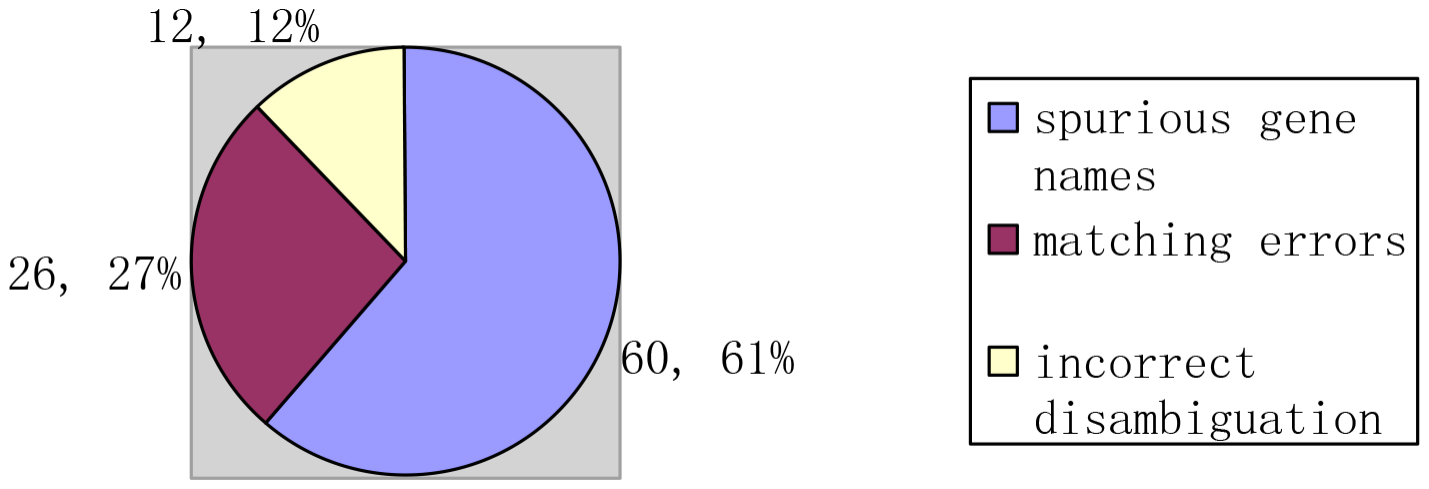
Causes for FP errors.

Analyzing the FN and FP errors in Table 1, we categorize the causes of errors as shown in Figure 6 and Figure 7.

The causes for false negatives are as follows (shown in Figure 6):

1. Mentions not identified. Gene mentions are not detected in the pre-processing (31 cases and 50% proportion), such as the enumeration “SMADs 1, 5 and 8” and “FA proteins A, C, G and F”. Also in some cases, the gene name is too long to be recognized, such as the mention “52-kD Ro/SSA lupus and Sjogren's syndrome auto-antigen”.
2. Mentions not matched. Gene mentions cannot get matched in the dictionary matching process because of the scale of lexicon (2 cases and 3% proportion). For example, “VP16” cannot get matched only using the lexicon provided by the BioCreative II dataset.
3. Incorrect disambiguation. Despite the useful context and extended semantic information are employed for disambiguation, the high similarity among the different identifiers still causes some errors (23 cases and 37% proportion), such as the mention “sp1”.
4. Erroneously filtered (6 cases and 10% proportion).

The causes for false positives are as follows (shown in Figure 7):

1. Spurious genes by our system (60 cases and 61% proportion), such as “TFIIIB”recognized as TP in one abstract while as FP in another.
2. Matching errors (26 cases and 27% proportion). For example, the gene mention “mitogen-activated protein kinase-interacting kinases 1” cannot get matched by using the exact matching and the false mapping with “mitogen activated protein kinase kinase kinase 1” is established by using the approximate matching.
3. Incorrect disambiguation errors (12 cases and 12% proportion).

## Conclusions

In this paper, we realize a multistage gene normalization system integrating different effective methods in each stage to complete the gene mention normalization task and make some comparisons among the results. Experimental results show that without using any external dictionary, our multistage system achieves an F-score of 90.1% on the BioCreative II GN test corpus, which is higher than that of most the state-of-the-art systems.
